# Immediate post-exercise blood pressure and arterial compliance in middle-aged and older normotensive females: A cross-sectional study

**DOI:** 10.1038/s41598-020-66104-8

**Published:** 2020-06-08

**Authors:** Eduardo C. Costa, Kevin F. Boreskie, D. Scott Kehler, David E. Kent, Jacqueline L. Hay, Rakesh C. Arora, Rodrigo A. V. Browne, Todd A. Duhamel

**Affiliations:** 1 Department of Physical Education, Federal University of Rio Grande do Norte, Natal, Brazil; 2 Faculty of Kinesiology & Recreation Management, University of Manitoba, Winnipeg, Canada; 3 Institute of Cardiovascular Sciences, St-Boniface Hospital Albrechtsen Research Centre, Winnipeg, Canada; 4 Division of Geriatric Medicine, Dalhousie University, Halifax, Canada; 5 Cardiac Sciences Program, St-Boniface Hospital Albrechtsen Research Centre, Winnipeg, Canada; 6 Department of Surgery, Max Rady College of Medicine, University of Manitoba, Winnipeg, Canada

**Keywords:** Physiology, Cardiology

## Abstract

This study examined whether immediate post-exercise systolic blood pressure (SBP) is associated with arterial compliance in middle-aged and older normotensive females. A total of 548 normotensive, non-frail females aged 55 years and older with no previous history of cardiovascular disease (CVD) participated in this cross-sectional study. Large and small arterial compliance were assessed by pulse wave analysis. Reduced arterial compliance was defined based on age and sex cutoffs. SBP was measured at rest and immediately following a 3-min moderate step-test. CVD risk factors were also assessed (e.g. resting systolic and diastolic BP, fasting glucose, triglycerides, cholesterol, body mass index). A total of 15.1% and 44.0% of the participants showed reduced large and small artery compliance, respectively. Immediate post-exercise SBP was associated with reduced large (OR 1.02 per 1 mmHg increase in post-exercise SBP, 95%CI 1.01–1.04; p = 0.010) and small (OR 1.02 per 1 mmHg increase in post-exercise SBP, 95%CI 1.00–1.03; p = 0.008) arterial compliance. Participants with highest immediate post-exercise SBP (quartile 4; i.e. ≥ 165 mmHg) showed increased odds ratios for reduced large (2.67, 95%CI 1.03–6.94; p = 0.043) and small (2.27, 95%CI 1.22–4.21; p = 0.010) arterial compliance compared to those with the lowest immediate post-exercise SBP (quartile 1; i.e. ≤ 140 mmHg), independent of other established CVD risk factors. Immediate post-exercise SBP following a brief moderate step-test seems to be able to discriminate reduced arterial compliance in middle-aged and older normotensive females.

## Introduction

Exaggerated exercise blood pressure (EEBP) responses to maximal exercise tests are associated with increased risk for myocardial dysfunction^1^, future cardiovascular disease (CVD)^2^ and hypertension^3^, as well as both CVD and all-cause mortality^2^. More recently, studies have shown that EEBP responses to brief light-moderate tests (i.e. ≤ 5 metabolic equivalents – METs) are also associated with future CVD events and mortality^4^, masked hypertension^5^, future hypertension^6^, impaired vascular health^7,8^, and impaired baroreflex sensitivity^9^. Thus, EEBP to even light-moderate tests could be used as a clinical tool to detect individuals at increased risk for CVD events. This is significant as light-moderate tests, unlike maximal tests, can be performed by most unfit and older individuals, and light-moderate intensity is perceived as less aversive by older adults as compared to maximal effort^10^.

Although the mechanisms underlying EEBP to brief light-moderate exercise are multifactorial and not completely understood, markers of impaired vascular health, including poor flow-mediated dilation, increased pulse wave velocity and decreased arterial compliance, are linked to EEBP at submaximal efforts^4,7,8^. Arterial compliance is one of the surrogate measures used to assess arterial stiffness, an important marker of vascular health due to its association with future CVD events and mortality^11^. This study examined whether a higher immediate post-exercise systolic BP following a 3-min moderate step-test would be associated with reduced arterial compliance in middle-aged and older normotensive females, independent of other established CVD risk factors. If confirmed, this information could be used to improve identification of females that may be at increased risk for future hypertension and CVD, even if they present with normal resting BP.

## Results

Table 1 shows the characteristics of the sample. A total of 548 middle-aged and older normotensive females were included in this study. Participants were primarily Caucasian (95%; n = 518). Approximately 80% (79.1%; n = 431) of the cohort received post-secondary education and 25.7% lived alone (n = 140). The prevalence of reduced large and small arterial compliance was 15.1% (n = 83) and 44.0% (n = 241), respectively.

**Table 1:** Characteristics of the participants (n = 548).

Variables	Median and percentiles 25–75
Age (years)	63 (60–68)
Body mass index (kg/m^2^)	25.0 (22.5–28.3)
Fasting glucose (mmol/L)	5.27 (4.97–5.61)
Triglycerides (mmol/L)	0.89 (0.69–1.26)
HDL-cholesterol (mmol/L)	1.83 (1.53–2.21)
LDL-cholesterol (mmol/L)	3.44 (2.81–4.05)
Total cholesterol (mmol/L)	5.48 (4.90–6.08)
Small arterial compliance (ml/mmHgx100)	3.9 (2.4–5.6)
Large arterial compliance (ml/mmHgx10)	12.5 (10.4–15.0)
Resting systolic BP (mmHg)	123 (115–131)
Resting diastolic BP (mmHg)	69 (63–75)
Resting pulse pressure (mmHg)	54 (48–59)
Immediate post-exercise systolic BP (mmHg)	153 (140–164)
Increase in immediate post-exercise systolic BP (mmHg)	29 (20–39)
6-minute walking test (m)	574 (540–615)
Pre-frailty (n, %)	244 (44.5%)
Current smokers (n, %)	10 (1.8%)
Ex-smokers (n, %)	211 (38.5%)
Diabetes medication (n, %)	5 (0.9%)
Lipid-lowering medication (n, %)	69 (12.6%)
Framingham Risk Score	
Low risk (<10%)	472 (86.1%)
Moderate risk (≥10% and <20%)	74 (13.5%)
High risk (≥20%)	2 (0.4%)

Abbreviations: BP, blood pressure.

Categorical variables are expressed as absolute (n) and relative (%) frequencies.

**Figure 1:**
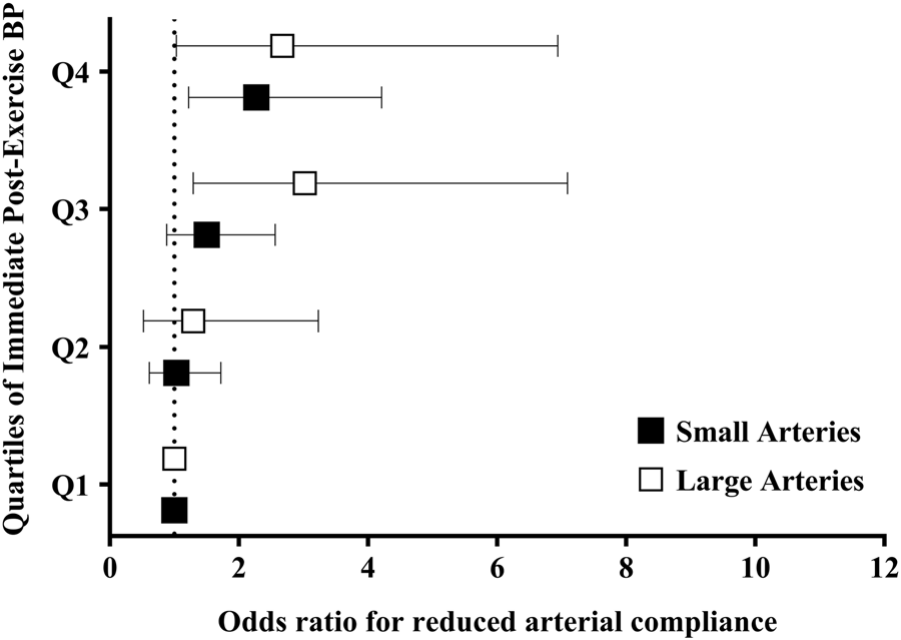
Odds ratio for reduced small and large arterial compliance according to quartiles of immediate post-exercise systolic blood pressure (n = 548). Quartile 1 (Q1): immediate post-exercise systolic BP ≤ 140 mmHg; Quartile 2 (Q2): immediate post-exercise systolic BP 141-153 mmHg; Quartile 3 (Q3): `–164; Quartile 4 (Q4): immediate post-exercise systolic BP ≥ 165 mmHg. *Different from Q1 (p < 0.05). ^a^Analysis adjusted for body mass index, resting systolic blood pressure, total cholesterol, 6-minute walking test, frailty status, and smoking. Goodness of fit of the model: p < 0.001 in Omnibus test and p = 0.837 in Hosmer-Lemeshow test. ^b^Analysis adjusted for age, body mass index, and resting systolic blood pressure. Goodness of fit of the model: p < 0.001 in Omnibus test and p = 0.344 in Hosmer-Lemeshow test.

Figure 1 and Supplementary Table 3 show the odds ratios for reduced small and large arterial compliance according to quartiles of immediate post-exercise BP. The participants of quartile 4 (i.e. immediate post-exercise BP ≥ 165 mmHg) showed higher odds for reduced small and large arterial compliance compared to those of quartile 1 (i.e. immediate post-exercise BP ≤ 140 mmHg; reference group). In addition, the participants of quartile 3 (i.e. immediate post-exercise BP 154–164 mmHg) showed higher odds for only reduced large arterial compliance compared to those of quartile 1. Supplementary Table 5 shows the odds ratios for reduced small and large arterial compliance according to quartiles of delta values (immediate post-exercise systolic BP – resting BP) of immediate post-exercise BP. No significant association was observed (p > 0.05).

**Figure 2:**
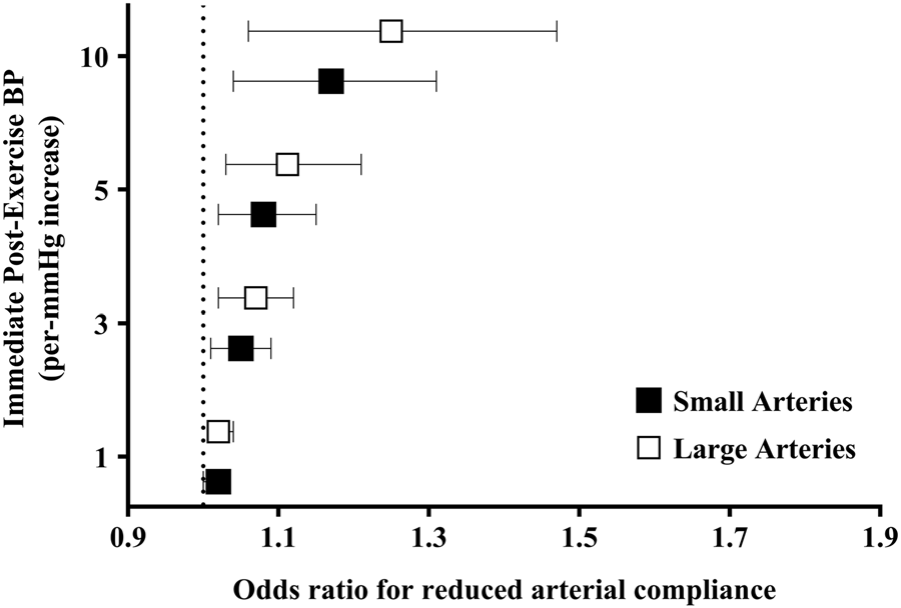
Odds ratio for reduced small and large arterial compliance according to per-mmHg increase in immediate post-exercise systolic blood pressure (n = 548). ^a^Analysis adjusted for body mass index, resting systolic blood pressure, total cholesterol, 6-minute walking test, frailty status, and smoking. Goodness of fit of the model: p < 0.001 in Omnibus test and p = 0.848 in Hosmer-Lemeshow test. ^b^Analysis adjusted for age, body mass index, and resting systolic blood pressure. Goodness of fit of the model: p < 0.001 in Omnibus test and p = 0.534 in Hosmer-Lemeshow test.

Figure 2 and Supplementary Table 4 shows the odds ratio for reduced small and large arterial compliance according to per-unit increase in immediate post-exercise BP. There was a linear association between per-unit increase in immediate post-exercise BP (for every 1, 3, 5, and 10 mmHg) and the odds ratio for reduced small and large arterial compliance. Supplementary Table 6 shows the odds ratios for reduced small and large arterial compliance according to per-unit increase in delta values of immediate post-exercise BP. No significant association was observed (p > 0.05).

In addition, it was tested the moderating effect of BMI on the association between immediate post-exercise BP and reduced arterial compliance. No moderating effect of BMI was observed for reduced small and large arterial compliance (p > 0.05).

Table 2 shows the differences among the participants from quartiles 1 to 4 in immediate post-exercise BP. Those with higher immediate post-exercise BP (quartile 4) were older, presented with a higher BMI, fasting glucose and triglycerides levels, resting systolic and diastolic BP, and pulse pressure, and had a higher prevalence of pre-frailty compared to the participants of lower immediate post-exercise BP (quartile 1). In addition, females with higher immediate post-exercise BP presented with lower HDL-cholesterol levels, small and large arterial compliance, and functional walking capacity as assessed by the 6-min walking test compared to those with lower immediate post-exercise BP (p < 0.05).

**Table 2:** Characteristics of the participants according to quartiles of immediate post-exercise systolic blood pressure (n = 548).

Variables	Quartile 1	Quartile 2	Quartile 3	Quartile 4	P value
N (%)	145 (26.4)	133 (24.3)	144 (26.3)	126 (23.0)	
Age (years)	61 (57–64)	63 (59–67)	65 (61–68)^a^	66 (62–71)^a,b^	<0.001
Body mass index (kg/m^2^)	23.9 (21.2–26.3)	24.1 (22.2–26.8)	25.4 (23.2–28.6)^a^	28.0 (24.6–31.6)^a,b,c^	<0.001
Fasting glucose (mmol/L)	5.14 (4.90–5.44)	5.20 (4.93–5.50)	5.32 (4.99–5.67)^a^	5.43 (5.04–5.84)^a,b^	<0.001
Triglycerides (mmol/L)	0.81 (0.67–1.18)	0.87 (0.68–1.24)	0.88 (0.69–1.15)	1.11 (0.81–1.61)^a,b,c^	<0.001
HDL-cholesterol (mmol/L)	1.96 (1.58–2.35)	1.84 (1.54–2.25)	1.81 (1.54–2.17)	1.71 (1.49–2.11)^a^	<0.001
LDL-cholesterol (mmol/L)	3.27 (2.66–3.97)	3.59 (2.85–4.14)	3.44 (2.97–3.95)	3.54 (2.83–4.04)	0.206
Total cholesterol (mmol/L)	5.36 (4.81–6.07)	5.52 (4.99–6.08)	5.39 (4.93–6.03)	5.53 (4.91–6.15)	0.381
Small arterial compliance (ml/mmHgx100)	4.7 (3.1–6.4)	4.2 (2.6–5.9)	3.4 (2.3–5.3)^a^	3.0 (2.1–5.0)^a,b^	<0.001
Large arterial compliance (ml/mmHgx10)	13.9 (11.6–16.9)	13.3 (11.3–15.2)	12.2 (9.5–14.8)^a,b^	11.5 (9.4–13.1)^a,b^	<0.001
Resting systolic BP (mmHg)	114 (108–120)	121 (115–127)^a^	126 (121–133)^a,b^	131 (125–135)^a,b^	<0.001
Resting diastolic BP (mmHg)	64 (60–68)	69 (64–74)^a^	71 (65–77)^a^	72 (67–76)^a,b^	<0.001
Resting pulse pressure (mmHg)	50 (44–54)	52 (46–56)	56 (51–60)^a,b^	57 (53–62)^a,b,c^	<0.001
Immediate post-exercise systolic BP (mmHg)	131 (124–135)	148 (144–151)^a^	160 (156–162)^a,b^	174 (168–185)^a,b,c^	<0.001
Increase in immediate post-exercise systolic BP (mmHg)	16 (10–20)	27 (20–33)^a^	32 (27–39)^a,b^	46 (39–59)^a,b,c^	<0.001
6-minute walking test (m)	600 (570–637)	600 (540–630)	570 (540–615)^a^	540 (502–585)^a,b,c^	<0.001
Pre-frailty (n, %)	56 (38.6%)	52 (39.1%)	61 (42.4%)	75 (59.5%)^a,b,c^	0.002
Ex-smokers/smokers (n, %)	51 (35.2%)	50 (37.6%)	64 (44.4%)	56 (44.4%)	0.271
Diabetes medication (n, %)	0 (0.0%)	0 (0.0%)	3 (2.1%)	2 (1.6%)	0.103
Lipid-lowering medication (n, %)	18 (12.4%)	11 (8.3%)	18 (12.5%)	22 (17.5%)	0.174

Abbreviations: BP, blood pressure; Quartile 1: immediate post-exercise systolic BP ≤ 140 mmHg; Quartile 2: immediate post-exercise systolic BP 141–153 mmHg; Quartile 3 immediate post-exercise systolic BP 154–164; Quartile 4: immediate post-exercise systolic BP ≥ 165 mmHg.

Data are expressed as median and percentiles 25–75 or absolute (n) and relative (%) frequencies.

^a^Different from quartile 1 (p < 0.05); ^b^Different from quartile 2 (p < 0.05); ^c^Different from quartile 3 (p < 0.05).

## Discussion

The main findings of this study were: (i) participants with the highest immediate post-exercise systolic BP (i.e. quartile 4) presented with increased odds ratios for reduced small and large arterial compliance compared to those with lowest immediate post-exercise systolic BP (quartile 1), even after adjusting for known CVD risk factors; (ii) there was a linear association between immediate post-exercise systolic BP and reduced small and large arterial compliance; (iii) the participants with the highest immediate post-exercise systolic BP presented with a different profile (e.g. older, higher body mass index, lower functional walking capacity) compared to the other participants.

Data from the Framingham Heart Study (n = 2115; 53% females; 59 ± 9 years) showed that exercise BP to stage 2 of the Bruce protocol was associated with increased arterial stiffness (i.e. carotid-femoral pulse wave velocity and brachial arterial blood flow velocity) and impaired endothelial function (i.e. flow-mediated dilation), independent of other CVD risk factors^8^. Previous studies have demonstrated that oscillatory or reflective arterial compliance may be a sensitive marker of endothelial dysfunction^12–14^. Gilani *et al*.^14^ have demonstrated that the inhibition of endothelial nitric oxide release by L-NAME decreases oscillatory or reflective arterial compliance (i.e. small arterial compliance). Thus, it seems possible that impaired vasodilation in response to a 3-min moderate step-test due to poor endothelial function may have been present in the participants with the highest immediate post-exercise systolic BP (i.e. quartile 4). In addition, it should be noted that the participants with the highest immediate post-exercise systolic BP also had a worse cardiometabolic profile (see Table 2), which is associated with a deleterious vascular remodeling, such as increased intima-media thickness in large arteries and increased media cross-sectional area and media/lumen ratio in small arteries^15^. Taken together, these alterations in vascular function may contribute to a higher systolic BP during daily activities performed at light-moderate intensities. This repetitive overload on the cardiovascular system, in turn, would contribute to further endothelial dysfunction and increased arterial stiffness.

In addition, Kokkinos *et al*.^16^ have demonstrated that, in middle-aged prehypertensive individuals, exercise systolic BP response to stage 1 of the Bruce protocol (i.e. 5 METs) was associated with left ventricular mass and left ventricular hypertrophy. The authors have shown that low-fit individuals have higher exercise systolic BP at this submaximal intensity, higher left ventricular mass and higher prevalence of left ventricular hypertrophy compared to moderate-fit and high-fit individuals. Similarly, Oh *et al*.^17^ have found that in normotensive individuals an EEBP to stage 1 of the Bruce protocol was associated with increased left ventricular mass. Therefore, higher exercise systolic BP to only a few minutes of moderate effort may discriminate non-hypertensive individuals at increased CVD risk, independent of high normal (120-139/80-89 mmHg)^16^ or optimal (<120/80 mmHg) resting BP levels^17^. Although we have measured systolic BP immediately following the 3-min moderate step-test, our results suggest that middle-aged and older normotensive females who present with the highest immediate post-exercise systolic BP may have a higher load on the heart and vasculature compared to those with the lowest immediate post-exercise systolic BP due to reductions in small and large arterial compliance. This, in turn, would increase their risk for future hypertension^6,18,19^. Future studies should confirm (or not) the assumption that immediate post-exercise systolic BP following a brief moderate intensity exercise test is associated with ventricular mass and whether it is able to discriminate individuals with ventricular hypertrophy.

An immediate post-exercise systolic BP ≥ 165 mmHg (i.e. quartile 4) was associated with increased odds ratio for reduced small and large arterial compliance and an exercise systolic BP between 154–164 mmHg (i.e. quartile 3) was associated with increased odds ratio for reduced large arterial compliance. To date, there is no specific threshold to determine EEBP to a moderate intensity exercise test (i.e. 5 METs). Previous studies have used exercise systolic BP values ranging from 130 to 180 mmHg to define an EEBP to moderate intensity exercise tests^5,8,9,16^. It should be noted that a threshold of 150 mmHg of exercise systolic BP at 5 METs has been associated with masked hypertension^5^, left ventricular hypertrophy^16^, and impaired baroreflex sensitivity^9^ in middle-aged and older individuals. Indeed, systolic BP measurement to a brief, moderate exercise test has some advantages compared to maximal exercise for discrimination of individuals with EEBP, such as: (i) it reflects the cardiovascular load of most daily activities^4^; (ii) it has greater accuracy due to decreased artefact noise^20^; (iii) moderate activities are less aversive than maximal efforts and can be performed by most unfit and unhealthy individuals^10^; (iv) it is time-efficient; (v) it is feasible at unsupervised settings for initial screening. However, it seems important to establish a more consistent threshold to define what is an EEBP to brief submaximal exercise tests and whether the timing of BP measurement (i.e. *during* or *immediately post-exercise*) could impact the ability of exercise systolic BP in discriminating individuals with reduced arterial compliance and other poor cardiovascular-related outcomes. Future studies should address this question.

Regarding the clinical implications of our findings, middle-aged and older normotensive females with high immediate post-exercise systolic BP following brief moderate intensity exercise tests should be identified for more comprehensive CVD risk screening, especially that which includes assessments of vascular function. Moreover, these individuals should be encouraged to make lifestyle changes that are known to reduce risk for CVD, such as performing regular physical activity, maintaining a healthy diet, and achieving weight reduction for those who are overweight or obese. Michishita *et al*.^21^ have shown that 12 weeks of a lifestyle modification program, including 60 minutes of moderate aerobic/dance exercise twice per week and counseling for healthy diet, decreased exercise BP at light, moderate, high, and maximal intensities in normotensive females with EEBP (peak systolic BP ≥ 190 mmHg; n = 25; 58.2 ± 10.3 years). Also, the authors showed that the reduction in exercise systolic BP was independently associated with improvements in arterial stiffness (i.e. brachial-ankle pulse wave velocity) and nitric oxide bioavailability (i.e. plasma nitrate/nitrite). It should be noted that high-fit individuals have lower exercise systolic BP response to moderate exercise than their low-fit peers, which suggests that improvements in cardiorespiratory fitness through regular physical activity can blunt an EEBP at submaximal intensities^16,22^.

This study has limitations that must be mentioned. First, this is a cross-sectional, secondary analysis of a prospective, observational cohort study. Therefore, it is not possible to establish causality for the observed associations between immediate post-exercise systolic BP and arterial compliance. In addition, the relationship between BP and arterial stiffness seems to be bidirectional, meaning that higher BP can damage the vasculature leading to increased arterial stiffness (or less compliant arteries), and similarly increased arterial stiffness can increase vascular pressure to precede high BP^18,23^. Second, the study included only middle-aged and older females and almost all participants were Caucasian (n = 518; 94.5%), which limits the generalizability of our findings to other females from different ethnic groups, males or population groups of different ages. Third, heart rate was not assessed during the step-test, which does not allow to confirm if a moderate intensity effort was reached by all participants. Fourth, immediate post-exercise BP may not have captured the peak exercise systolic BP. On the other hand, this procedure may have increased the accuracy of BP measurement due to decreased artefact and standardized position of the participants.

## Conclusion

Immediate post-exercise systolic BP following a 3-min moderate intensity step-test seems to be able to discriminate reduced arterial compliance in middle-aged and older normotensive females, independent of other established CVD risk factors and despite normal resting BP. Longitudinal studies should investigate whether middle-aged and older normotensive females with high immediate post-exercise systolic BP will have higher incidence of hypertension, cardiovascular diseases and/or events compared to the other females. As the original observational trial^24^ will be completed in 2022, it will be possible to test this hypothesis in the future.

## Methods

### Study design

The Strengthening the Reporting of Observational Studies in Epidemiology (STROBE) statement for cross-sectional studies was followed in the development of this manuscript^25^. This study is a cross-sectional, secondary analysis of a prospective, observational cohort study designed to determine the efficacy of a series of non-invasive procedures to identify middle-aged and older females, who are at elevated risk for CVD over a five-year period^24^. Briefly, this screening protocol is based, in part, on the measures of i) resting BP, ii) BP immediately following a 3-min moderate step-test (5 METs), iii) large arterial compliance and iv) small arterial compliance. The predictive value of this alternative CVD screening will be compared to the Framingham Risk Score^26^ in order to determine if one method has better sensitivity for estimating risk for CVD^24^.

### Ethical approval

This study was approved by the Education/Nursing Research Ethics Board at the University of Manitoba (#E2016:095-HS2035) and registered publicly (ClinicalTrials.gov Identifier: NCT0286321). The study was conducted according to the Declaration of Helsinki. The participants were informed about all procedures of the study and provided written informed consent.

### Participants

Data from the participants included in this cross-sectional analysis were obtained from the database of the original prospective, observational cohort study^24^. Inclusion criteria for this study were: i) females aged 55 years and older; (ii) non-frail according to the Fried frailty score^27^; (iii) no self-reported previous hospitalization for ischemic heart disease, acute myocardial infarction, stroke, percutaneous coronary intervention, coronary arterial bypass surgery, congestive heart failure, and peripheral arterial disease; (iv) no previous diagnosis of hypertension according to the Hypertension Canada’s 2018 Guidelines for Diagnosis, Risk Assessment, Prevention, and Treatment of Hypertension in Adults and Children^28^; (v) resting BP lower than 140/90 mmHg.

### Assessment of arterial compliance

The methods for BP and arterial compliance measurements were detailed previously^24^. The participants rested for 10 minutes in a supine position in a controlled room (24-26 °C degrees) before resting BP and arterial compliance measurements were taken. A BP cuff, attached to the HD/PulseWave CR-2000 Research CardioVascular Profiling System, was placed on the participant’s left arm while they were in a supine position. The BP cuff size was adapted to the circumference of the arm of each participant according to the manufacturer’s recommendations. Large and small arterial compliance were assessed using computerized radial arterial pulse wave analysis (HD/PulseWave CR-2000 Research CardioVascular Profiling System, Hypertension Diagnostics, Eagan, Minnesota, USA). To measure arterial compliance, a wrist stabilizer was placed on the participant’s right wrist to minimize wrist movement in order to facilitate the analysis of radial pulsation by the sensor. A pulse wave sensor was then placed on the location of strongest pulsation of the radial artery. The sensor was adjusted until achieving the highest relative signal strength before initiating the test. Radial artery wave forms were recorded for 30 seconds with an arterial tonometer sensor. The HD/PulseWave CR-2000 Research CardioVascular Profiling System assesses the diastolic decay and waveform transmitted to it through the sensor. Based on the modified Windkessel model, the device can then determine the large artery elasticity index (capacitive arterial compliance, which is the marker of large arterial compliance) and the small artery elasticity index (oscillatory or reflective arterial compliance, which is the marker of small arterial compliance). This method has been validated with invasive testing. Briefly, arterial compliance calculated from noninvasive waveform and noninvase cardiac output algorithms was compared with arterial compliance calculated from brachial arterial invasive pressure measurents and the indocyanine green dye dilution cardiac output measurement. Capacitive arterial compliance measured noninvasively showed a significant correlation with the invasive measure (r = 0.82; p < 0.001) with a mean difference (invasive – noninvasive) of −0.34 ± 0.36 mL/mmHg. Oscillatory arterial compliance measured noninvasively also showed a significant correlation with the invasive measure (r = 0.62; p < 0.001) with a mean difference (invasive – noninvasive) of −0.018 ± 0.025 mL/mmHg^29^. A multicenter study including healthy individuals from 15 to 80 years of age showed that intra- and inter-visit arterial compliance measures differed by less than 3% and 4%, respectively, with high reproducibility^30^. Reduced arterial compliance was defined as borderline and/or abnormal values based on age and sex cutoffs^31^. For females under 65 years, values considered reduced arterial compliance were equal to or lower than 9.9 mL/mmHg × 10 and 3.9 mL/mmHg × 100 for large and small arteries, respectively. For females over the age of 65 years, values considered reduced arterial compliance were equal to or lower than 8.9 mL/mmHg × 10 and 2.9 mL/mmHg × 100 for large and small arteries, respectively^31^. Previous studies have shown that reduced small arterial compliance, as assessed by the HD/PulseWave CR-2000 Research CardioVascular Profiling System, predicts future adverse cardiovascular events^31,32^.

### Resting and immediate post-exercise blood pressure measures

The HD/PulseWave CR-2000 Research CardioVascular Profiling System measures BP by oscillometry. Resting BP and arterial compliance measurements were conducted with the participants in a supine position following 10 minutes of rest. Afterward, the participants were instructed to perform a 3-min moderate step-test on a two-step stool according to the Dundee Step Test (each step is 20.3 centimeters tall; cadence: 23 steps/min or 92 beats/min guided by a metronome)^33^, which is equivalent to 5 METs and represents a physiological demand commonly imposed in daily activities. Each complete step consists of four movements (going up and down on the two-step stool) and each of them is timed using the metronome. Immediately following the step-test, the participants walked ~5 meters, lied down and had their BP measured once again in a supine position. The post-exercise BP measure occurred within 30 s after the step-test. All procedures were established previously for the original observational study^24^.

### Statistical analysis

Normal data distribution was verified using the Kolmogorov-Smirnov test and normal Q-Q plot. Data was non-normally distributed and therefore continuous variables were expressed as median and interquartile range (25^th^ and 75^th^ percentile). Categorical data were expressed as absolute and relative rates. Kruskal-Wallis test was used to compare the immediate post-exercise BP quartiles. Mann-Whitney U, Chi-squared and Fisher’s exact tests were used for the bivariate analysis. Binomial logistic regression was applied to the multivariate analyses. The odds ratios (OR) and corresponding 95% confidence intervals (CI) were calculated for reduced small and large arterial compliance using the following predictors (models): (a) quartiles of immediate post-exercise BP; (b) per-unit increase every 1 mmHg in immediate post-exercise BP; (c) per-unit increase every 3 mmHg in immediate post-exercise BP; (d) per-unit increase every 5 mmHg in immediate post-exercise BP; and (e) per-unit increase every 10 mmHg in immediate post-exercise BP. All multivariate models were adjusted for the independent variables that were different between the groups with and without reduced small and large arterial compliance at p < 0.20 (see Supplementary Table 1 for reduced small arterial compliance and Supplementary Table 2 for reduced large arterial compliance). The independent variables that showed a p value < 0.10 remained in the adjusted model (Backward method). The following variables were considered as potential confounders: age, smoking status, body mass index, resting BP, total cholesterol, HDL-cholesterol, LDL-cholesterol, triglycerides, fasting glucose, lipid lowering medication(s), diabetes lowering medication(s), 6-min walking test, and the Fried frailty phenotype. The methodological procedures related to the measurement of these abovementioned variables have been described previously^24^. The assumptions of binomial logistic regression were tested, in all models, including: (a) the linear relationship between the continuous independent variables and the logit transformation of the dependent variable; (b) multicollinearity; (c) significant outliers, high leverage points or highly influential points. The Hosmer-Lemeshow and Omnibus tests were performed to assess the goodness of fit for the models. The level of significance was set at p < 0.05. Statistical procedures were performed using IBM SPSS Statistics for Win/v.25.0 (IBM Corp., Armonk, NY).

## Supplementary information

Supplementary Tables.
