# Supplementary material for: Immediate post-exercise blood pressure and arterial compliance in middle-aged and older normotensive females: A cross-sectional study
Source: Sci Rep. 2020 Jun 8;10:9205. doi: 10.1038/s41598-020-66104-8 (PMC7280524; doi:10.1038/s41598-020-66104-8)
Supplement: Supplementary file 1 — Supplementary Tables. [file 41598_2020_66104_MOESM1_ESM.docx]

**Immediate post-exercise blood pressure and arterial compliance in middle-aged and older normotensive females: A cross-sectional study**

**Eduardo C. Costa^1,*^, Kevin F. Boreskie^2,3^, Dustin S. Kehler^2,3,4^, David E. Kent^2,3^, Jacqueline L. Hay^2,3^, Rakesh C. Arora^3,5,6^, Rodrigo A.V. Browne^1^ & Todd A. Duhamel^2,3,5^**

^1^Department of Physical Education, Federal University of Rio Grande do Norte, Natal, Brazil

^2^Health, Leisure & Human Performance Research Institute, Faculty of Kinesiology & Recreation Management, University of Manitoba, Winnipeg, Canada

^3^Institute of Cardiovascular Sciences, St-Boniface Hospital Albrechtsen Research Centre, Winnipeg, Canada

^4^Division of Geriatric Medicine, Dalhousie University, Halifax, Canada

^5^Cardiac Sciences Program, St-Boniface Hospital Albrechtsen Research Centre, Winnipeg, Canada

^6^Department of Surgery, Max Rady College of Medicine, University of Manitoba, Winnipeg, Canada

*[ecc@ufrnet.br](mailto:ecc@ufrnet.br)

**Supplementary Table** **1**. Bivariate analysis of the participants with and without reduced small arterial compliance (n = 548).

| **Variables** | **Reduced Small Arterial Compliance** | | **P value** |
| --- | --- | --- | --- |
|  | **Yes** | **No** |  |
| N (%) | 241 (44%) | 307 (56%) |  |
| Age (years) | 64 (60–68) | 63 (59–68) | 0.042 |
| Body mass index (kg/m^2^) | 24.3 (21.9–27.7) | 25.6 (23.2–28.8) | 0.001 |
| Fasting glucose (mmol/L) | 5.26 (4.96–5.63) | 5.29 (4.98–5.60) | 0.815 |
| Triglycerides (mmol/L) | 0.86 (0.66–1.26) | 0.93 (0.71–1.27) | 0.339 |
| HDL-cholesterol (mmol/L) | 1.86 (1.60–2.29) | 1.80 (1.50–2.19) | 0.047 |
| LDL-cholesterol (mmol/L) | 3.44 (2.91–4.12) | 3.45 (2.76–4.02) | 0.222 |
| Total cholesterol (mmol/L) | 5.52 (5.02–6.12) | 5.40 (4.77–5.99) | 0.030 |
| Resting systolic BP (mmHg) | 126 (119–132) | 121 (113–129) | <0.001 |
| Resting diastolic BP (mmHg) | 70 (65–76) | 67 (62–73) | <0.001 |
| Resting pulse pressure (mmHg) | 55 (50–61) | 52 (48–57) | 0.001 |
| Immediate post-exercise systolic BP (mmHg) | 156 (143–165) | 151 (136–162) | 0.001 |
| Increase in immediate post-exercise systolic BP (mmHg) | 30 (19–40) | 29 (20–39) | 0.825 |
| 6-minute walking test (m) | 570 (525–615) | 585 (540–630) | 0.032 |
| Pre-frailty (n, %) | 128 (53.1%) | 116 (37.8%) | <0.001 |
| Ex-smokers/smokers (n %) | 109 (45.2%) | 112 (36.5%) | 0.015 |
| Diabetes medication (n, %) | 4 (1.7%) | 1 (0.3%) | 0.175 |
| Lipid-lowering medication (n, %) | 28 (11.6%) | 41 (13.4%) | 0.543 |

Abbreviations: BP, blood pressure.

Data are expressed as median and percentiles 25-75.

**Supplementary Table** **2**. Bivariate analysis of the participants with and without reduced large arterial compliance (n = 548).

| **Variables** | **Reduced Large Arterial Compliance** | | **P value** |
| --- | --- | --- | --- |
|  | **Yes** | **No** |  |
| N (%) | 83 (15.1%) | 465 (84.9%) |  |
| Age (years) | 67 (62–72) | 63 (59–67) | <0.001 |
| Body mass index (kg/m^2^) | 24.1 (21.5–27.1) | 25.2 (22.6–28.4) | 0.024 |
| Fasting glucose (mmol/L) | 5.28 (5.03–5.57) | 5.26 (4.96–5.62) | 0.494 |
| Triglycerides (mmol/L) | 0.96 (0.71–1.21) | 0.88 (0.69–1.27) | 0.408 |
| HDL-cholesterol (mmol/L) | 1.88 (1.59–2.18) | 1.82 (1.53–2.23) | 0.478 |
| LDL-cholesterol (mmol/L) | 3.40 (2.88–3.91) | 3.45 (2.80–4.08) | 0.766 |
| Total cholesterol (mmol/L) | 5.44 (4.90–6.22) | 5.48 (4.91–6.05) | 0.636 |
| Resting systolic BP (mmHg) | 130 (124–135) | 122 (115–129) | <0.001 |
| Resting diastolic BP (mmHg) | 70 (63–76) | 68 (63–74) | 0.249 |
| Resting pulse pressure (mmHg) | 57 (54–65) | 53 (47–58) | <0.001 |
| Immediate post-exercise systolic BP (mmHg) | 161 (148–168) | 151 (137–163) | <0.001 |
| Increase in immediate post-exercise systolic BP (mmHg) | 30 (22–42) | 29 (19–39) | 0.142 |
| 6-minute walking test (m) | 570 (510–630) | 585 (540–615) | 0.024 |
| Pre-frailty (n, %) | 42 (50.6%) | 202 (43.4%) | 0.227 |
| Ex-smokers/smokers (n %) | 34 (41%) | 187 (40.2%) | 0.503 |
| Diabetes medication (n, %) | 0 (0%) | 5 (1.1%) | 1.000 |
| Lipid-lowering medication (n, %) | 14 (16.9%) | 55 (11.8%) | 0.202 |

Abbreviations: BP, blood pressure.

Data are expressed as median and percentiles 25-75.

**Supplementary Table 3.** Odds ratio for reduced small and large arterial compliance according to quartiles of immediate post-exercise systolic blood pressure (n = 548).

|  | **Reduced Small Arterial Compliance** | | |  | **Reduced Large Arterial Compliance** | | |
| --- | --- | --- | --- | --- | --- | --- | --- |
|  | **OR^a^** | **(95% CI)** | **P value** |  | **OR^b^** | **(95% CI)** | **P value** |
| **Immediate Post-Exercise Systolic BP**  **(per quartile)** |  |  |  |  |  |  |  |
| Q1 ≤ 140 mmHg | 1.00 | (Reference) |  |  | 1.00 | (Reference) |  |
| Q2 141-153 mmHg | 1.03 | (0.61 to 1.72) | 0.926 |  | 1.29 | (0.52 to 3.23) | 0.583 |
| Q3 154-164 mmHg | 1.50 | (0.88 to 2.56) | 0.133 |  | 3.02 | (1.29 to 7.09) | 0.011 |
| Q4 ≥ 165 mmHg | 2.27 | (1.22 to 4.21) | 0.010 |  | 2.67 | (1.03 to 6.94) | 0.043 |

Abbreviations: OR, odds ratio; CI, confidence interval; BP, blood pressure; Q, quartile.

^a^Analysis adjusted for body mass index, resting systolic blood pressure, total cholesterol, 6-minute walking test, frailty status, and smoking. Goodness of fit of the model: p < 0.001 in Omnibus test and p = 0.837 in Hosmer-Lemeshow test. ^b^Analysis adjusted for age, body mass index, and resting systolic blood pressure. Goodness of fit of the model: p < 0.001 in Omnibus test and p = 0.344 in Hosmer-Lemeshow test.

**Supplementary Table 4.** Odds ratio for reduced small and large arterial compliance according to per-mmHg increase in immediate post-exercise systolic blood pressure (n = 548).

|  | **Reduced Small Arterial Compliance** | | |  | **Reduced Large Arterial Compliance** | | |
| --- | --- | --- | --- | --- | --- | --- | --- |
|  | **OR^a^** | **(95% CI)** | **P value** |  | **OR^b^** | **(95% CI)** | **P value** |
| **Immediate Post-Exercise Systolic BP**  **(per-mmHg increase)** |  |  |  |  |  |  |  |
| 1 mmHg | 1.02 | (1.00; 1.03) | 0.008 |  | 1.02 | (1.01; 1.04) | 0.010 |
| 3 mmHg | 1.05 | (1.01; 1.09) | 0.008 |  | 1.07 | (1.02; 1.12) | 0.010 |
| 5 mmHg | 1.08 | (1.02; 1.15) | 0.008 |  | 1.12 | (1.03; 1.21) | 0.010 |
| 10 mmHg | 1.17 | (1.04; 1.31) | 0.008 |  | 1.25 | (1.06; 1.47) | 0.010 |

Abbreviations: OR, odds ratio; CI, confidence interval; BP, blood pressure.

^a^Analysis adjusted for body mass index, resting systolic blood pressure, total cholesterol, 6-minute walking test, frailty status, and smoking. Goodness of fit of the model: p < 0.001 in Omnibus test and p = 0.848 in Hosmer-Lemeshow test. ^b^Analysis adjusted for age, body mass index, and resting systolic blood pressure. Goodness of fit of the model: p < 0.001 in Omnibus test and p = 0.534 in Hosmer-Lemeshow test.

**Supplementary Table 5.** Odds ratio for reduced small and large arterial compliance according to quartiles of delta values of immediate post-exercise systolic blood pressure (n = 548).

|  | **Reduced Small Arterial Compliance** | | |  | **Reduced Large Arterial Compliance** | | |
| --- | --- | --- | --- | --- | --- | --- | --- |
|  | **OR^a^** | **(95% CI)** | **P value** |  | **OR^b^** | **(95% CI)** | **P value** |
| **∆ Immediate Post-Exercise Systolic** **BP**  **(per quartile)** |  |  |  |  |  |  |  |
| Q1 ≤ 20 mmHg | 1.00 | (Reference) |  |  | 1.00 | (Reference) |  |
| Q2 21-29 mmHg | 1.17 | (0.70 to 1.96) | 0.557 |  | 1.46 | (0.71 to 3.00) | 0.305 |
| Q3 30-39 mmHg | 1.24 | (0.75 to 2.04) | 0.397 |  | 1.00 | (0.47 to 2.11) | 0.997 |
| Q4 ≥ 40 mmHg | 1.22 | (0.71 to 2.09) | 0.481 |  | 2.06 | (0.98 to 4.34) | 0.056 |

Abbreviations: OR, odds ratio; CI, confidence interval; BP, blood pressure; Q, quartile; ∆ = delta of change (immediate post-exercise systolic blood pressure – resting systolic blood pressure).

^a^Analysis adjusted for body mass index, resting systolic blood pressure, total cholesterol, 6-minute walking test, frailty status, and smoking. Goodness of fit of the model: p < 0.001 in Omnibus test and p = 0.902 in Hosmer-Lemeshow test. ^b^Analysis adjusted for age, body mass index, and resting systolic blood pressure. Goodness of fit of the model: p < 0.001 in Omnibus test and p = 0.554 in Hosmer-Lemeshow test.

**Supplementary Table 6.** Odds ratio for reduced small and large arterial compliance according to delta values of immediate post-exercise systolic blood pressure (n = 548).

|  | **Reduced Small Arterial Compliance** | | |  | **Reduced Large Arterial Compliance** | | |
| --- | --- | --- | --- | --- | --- | --- | --- |
|  | **OR^a^** | **(95% CI)** | **P value** |  | **OR^b^** | **(95% CI)** | **P value** |
| **∆ Immediate Post-Exercise Systolic BP (mmHg)** | 1.00 | (0.99; 1.02) | 0.619 |  | 1.01 | (0.99; 1.03) | 0.162 |

Abbreviations: OR, odds ratio; CI, confidence interval; BP, blood pressure; ∆ = delta of change (immediate post-exercise systolic blood pressure – resting systolic blood pressure).

^a^Analysis adjusted for body mass index, resting systolic blood pressure, total cholesterol, 6-minute walking test, frailty status, and smoking. Goodness of fit of the model: p < 0.001 in Omnibus test and p = 0.811 in Hosmer-Lemeshow test. ^b^Analysis adjusted for age, body mass index, and resting systolic blood pressure. Goodness of fit of the model: p < 0.001 in Omnibus test and p = 0.311 in Hosmer-Lemeshow test.
